# Protocol for Co-Design, Development, and Open Trial of a Prototype Game-Based eHealth Intervention to Treat Anxiety in Young People With Long-Term Physical Conditions

**DOI:** 10.2196/resprot.7250

**Published:** 2017-09-22

**Authors:** Hiran Thabrew, Karolina Stasiak, Sally Merry

**Affiliations:** ^1^ University of Auckland Department of Psychological Medicine Auckland New Zealand

**Keywords:** long-term physical conditions, chronic illness, anxiety, eHealth, gaming, young people, treatment, cognitive behavior therapy, biofeedback

## Abstract

**Background:**

Approximately 10% to 12% of New Zealand young people (and 21% of Maori young people) have long-term physical conditions and are more likely to develop psychological problems, particularly anxiety and depression. Delayed treatment leads to worse management of physical conditions, school absence, and poorer long-term outcomes. Recently, electronic health (eHealth) interventions have been shown to be as good as face-to-face therapy and biofeedback techniques have been shown to enhance relaxation during the treatment of anxiety. In addition, young people with long-term physical conditions have expressed a preference for more technologically based support, including game-based interventions, to deal with psychological issues, particularly anxiety.

**Objective:**

The aim of this study is to develop a prototype game-based eHealth intervention to address anxiety in young people with long-term physical conditions. The game will be based on the principles of cognitive behavior therapy (CBT) and will integrate a module of biofeedback-based relaxation.

**Methods:**

During the first phase of the study, up to 48 young people with long-term physical conditions aged 13 to 18 years, attending a tertiary pediatric hospital will be invited to participate in a 3-stage series of co-design workshops. Following the design, development, and refinement of a working prototype, during the second phase of the study, a further 20 young people with long-term physical conditions and anxiety will be recruited from the same location to participate in an open pilot trial to evaluate its acceptability, usability, and preliminary efficacy.

**Results:**

Changes in anxiety will be measured using the Generalized Anxiety Disorder 7-item scale (GAD-7) and the Spence Child Anxiety Scales (SCAS) at the end of every module (recommended to be completed weekly), post intervention, and 3 months later. Usability of the intervention will be measured using the System Usability Scale (SUS) and by measuring frequency and quantity of use of the intervention. Acceptability of the intervention will be assessed using brief, open-ended questionnaires and semi-structured interviews, the data from which will be analyzed using a general inductive approach. Recruitment to the study commenced in January 2017 and data collection will be completed by the end of December 2017.

**Conclusions:**

If acceptable and useful, this game-based eHealth intervention may offer a cost-effective and clinically useful intervention for addressing the psychological needs of over 16,000 young people with long term health conditions in New Zealand.

**Trial Registration:**

Australian New Zealand Clinical Trials Network Registry (ANZCTR): ACTRN12616001253493p; https://www.anzctr.org.au/Trial/Registration/TrialReview.aspx?id=371443 (Archived by WebCite at http://www.webcitation.org/6sYB716lf)

## Introduction

Long-term physical conditions are increasingly common in children and young people and those that last more than 3 months and impair functioning affect 10% to 12% of children globally [1]. These conditions include asthma, diabetes, epilepsy, and obesity, among others [2,3]. The prevalence of long-term physical conditions in childhood is increasing [4] and in some high-income countries, due to improvements in hygiene, immunization, and access to medical care, it is greater than that of acute illness [5].

Psychological problems, especially anxiety, are more likely in these individuals such that long-term physical conditions in children have consistently been associated with an increased risk of psychological problems [6-11], particularly anxiety which has been identified in up to 40% of these individuals [12]. The likelihood of anxiety is related to young people’s developmentally linked internal ability to manage stress, to family factors, and the cumulative allostatic load-related impact of illness and treatment, particularly distress and pain [13,14]. Anxiety and other psychological problems can occur during or even following the completion of medical (eg, cancer) treatment [15] and may be associated with school absence, poor academic performance, and lower health-related quality of life [16,17]. Left untreated, anxiety disorders tend to have a chronic and unremitting course [18] and tend to increase the risk for adult psychiatric disorders, including depression and substance use disorders [19].

Access to, and effectiveness of, treatments for these problems are currently limited. Psychological problems are traditionally addressed using psychotherapies such as cognitive behavior therapy (CBT) and pharmacotherapy (eg, anxiolytic or antidepressant medication). There is limited evidence that these therapies are effective for children with long-term physical conditions [20]. Psychotherapies are often not accessible and although 60% of children with anxiety who receive CBT show an adequate treatment response [21], there does appear to be room for improving current treatment.

With the increasing popularity of smart technology, release of app-based interventions, and calls from international organizations such as The Lancet Global Mental Health Group [22] for the introduction of innovative and accessible cognitive and behavioral strategies to treat anxiety, depressive, and other common mental health problems, electronic health (eHealth) interventions may have a useful role to play in addressing common mental health problems in young people with long-term physical conditions. Health games, such as Smart, Positive, Active, Realistic, X-factor Thoughts (SPARX) [23], have already been shown to be as good as face to face treatment for addressing depression in young people. Similar interventions such as Body Signs, Relaxation, Active Helpful Thoughts, Victory Over Your Fears, Enjoy (BRAVE) online [24] exist to treat anxiety but are not focused on health-related anxieties and are not widely available outside Australia. During a recent investigation by Thabrew and colleagues, young people with long-term physical conditions identified anxiety as the most significant psychological issue that they face. Together with their families and clinicians, they confirmed limited knowledge of and access to eHealth interventions and expressed support for the development of eHealth interventions targeted towards their needs [25].

Traditional psychological therapies often include a component of psychologically or chemically induced relaxation and there is increasing evidence that newer, more technologically-based forms of therapy, such a biofeedback, may achieve similar results, either alone or in combination with traditional therapies [26]. Furthermore, some biofeedback interventions have already been combined with game-based technology to reduce stress or treat behavior disorders [27]. Biofeedback involves the use of electrical or electro-mechanical equipment to measure physiologic processes occurring in a person and then feed this information back to them to develop a greater awareness and ability to control changes within their bodies with and without equipment [28] and improve health and performance [29]. There are a number of types of biofeedback including heart rate variability (HRV), electroencephalography (EEG), and pneumography (PNG). HRV is already used by some pediatric mental health teams in New Zealand (personal communication from Dr Louise Webster, Starship Hospital, Auckland) and a recent systematic review by Dr Thabrew supported further research into HRV biofeedback as a treatment for anxiety (in press).

The aims of this study are (1) to develop a prototype game-based eHealth intervention for treating anxiety in young people with long-term physical conditions via co-design with end-users at Starship Hospital; (2) to evaluate the acceptability of this intervention with its intended audience; (3) to evaluate the utility of this intervention with its intended audience; (4) to evaluate the efficacy of this intervention in a preliminary manner; and (5) to evaluate the feasibility of delivery of this intervention with its intended audience.

## Methods

### Research Strategy

The study will employ a mixed-methods design to co-design, produce, and test a prototype game-based eHealth intervention for treating anxiety in young people with long-term physical conditions.

### Study Design

The study will involve 2 phases. During the first phase, a prototype game-based eHealth intervention will be designed and refined via a co-design process with young people with long-term physical conditions attending a tertiary pediatric hospital in Auckland, New Zealand. Three stages of iterative consultation are planned, with 4 focus groups of up to 12 participants at each stage. At least one focus group at each stage will be arranged for Māori young people to ensure that, in the spirit of biculturalism, the intervention is culturally acceptable to them. By the end of this phase, a working prototype that is ready for pilot testing will be created and refined to ensure it is compatible with end-user expectations. During the second phase, an open pilot trial will be undertaken with 20 young people with long-term physical conditions and anxiety from the same hospital. Each will be loaned a portable device (iPad or similar tablet) on which the prototype intervention, questionnaires, and scales will be preloaded. They will be given up to 8 weeks to complete the intervention at a speed and frequency of their choosing.

### Study Population

Up to 48 young people with long-term physical conditions who are either inpatients or outpatients attending a tertiary pediatric hospital in Auckland, New Zealand will participate in focus groups during each stage of the first phase. Following this, 20 young people will then participate in the second phase, open trial.

#### Inclusion Criteria

Young people will be included in the first phase of the study if they are aged between 13 to 18 years, have any long-term physical condition over 3 months duration (eg, asthma, diabetes, cancer, cystic fibrosis), are of any ethnicity, do and do not have a known anxiety disorder, do or do not have any co-morbid mental health condition, can intellectually and physically use the device and intervention, and if they understand English and are able to provide informed consent or assent. Young people will be included in the second phase of the study if they meet all of the above criteria and have any symptoms of anxiety (not necessarily a diagnosed anxiety disorder).

#### Exclusion Criteria

Young people will be excluded from participation if they do not meet all of the inclusion criteria, if they have an intellectual disability or cannot speak English, or if they have recently undertaken or are undertaking CBT or other forms of psychotherapy, biofeedback therapy, or pharmacotherapy with anxiolytic medication, as these may confound the effectiveness of the prototype game-based eHealth intervention.

### Intervention

The prototype game-based eHealth intervention that will be co-designed with young people with long-term physical conditions will be a 4 to 8 module online, game-based intervention. Its content will be based on the principles of CBT and include an integrated or associated biofeedback-based relaxation component. It is anticipated that modules will take between 30 to 60 minutes each to be completed. Key elements that will be included are education about anxiety and coping strategies for anxiety, using one’s body (relaxation strategies), mind (recognizing unhelpful thoughts and cognitive restructuring), and actions (including graded exposure) to beat anxiety. The precise format and look of the prototype game-based eHealth intervention will be designed in conjunction with end users. The prototype intervention will be stored on a portable tablet, rather than available online or via a mobile app. Due to the co-design process that is being planned, the precise form and content of the intervention is not yet fully defined. However, key CBT-based principles including psychoeducation, relaxation, graded exposure to feared stimuli, and cognitive restructuring will be included. Further refinement of the intervention is likely on the basis of feedback from the pilot trial.

### Outcome Measures

The primary outcomes of the study are (1) acceptability of the prototype intervention, (ie, is the content and format acceptable to users), as assessed via a semi-structured interview following completion of the study at 8 weeks; (2) utility of the intervention (ie, is it useful), as assessed using the System Utility Scale (SUS) [30] following completion of the intervention at 8 weeks; and (3) feasibility of the intervention (ie, is it easy to deliver in a hospital/home setting), as assessed via a semi-structured interview following completion of the study at 8 weeks.

The secondary outcome of the study is efficacy (ie, does the intervention reduce anxiety and related issues). This will be assessed by measuring changes over time in the Generalized Anxiety Disorder, 7-item (GAD-7) [31], Spence Children’s Anxiety Scale (SCAS) [32], and the Pediatric Quality of Life Inventory (PedsQL) [33], as outlined in the schedule below (Table 1). The GAD-7 is a newer, brief scale for measuring anxiety that may be useful to incorporate into the final version of the intervention for ease of completion. The SCAS is a well-validated, 46-item self-report scale measuring child anxiety via an overall score and 6 subscales for panic/agoraphobia, social phobia, separation anxiety, obsessions/compulsions, fear of physical injury, and generalized anxiety. It has sound psychometric properties with internal consistency reported at 0.92 for the total child score. The PedsQL is a well-validated, 23-item self or parent report scale measuring quality of life. It has good internal consistency (0.88 for total scale), validity, and acceptability. It reliably distinguishes between healthy children and those with acute or long-term physical conditions.

### Statistical Methodology

Quantitative data will be analyzed using Microsoft Excel and Statistical Software Package (SPSS). Analyses will include basic descriptive statistics (eg, number of sessions completed, number of times device accessed, duration of use, changes in anxiety score, and demographic characteristics of the sample). McNemar’s chi-square tests and *t* tests will be used to assess the statistical significance of changes in anxiety scores over time. *P* values of less than .05 will be taken to indicate statistical significance and 95% confidence intervals will be used to establish the extent of any difference between pre- and post-measures. A sample size of 20 will enable detection of changes within the study group with effect sizes of 0.65 or more as statistically significance (alpha .05 with 80% power). Qualitative data will be analyzed using a general inductive approach. Collated text analyzed to identify emerging themes, which will then be independently coded by one researcher and a subset of 30% of results, will be cross-coded. NVIVO software will be used to handle transcripts.

**Table 1:** Schedule of assessment procedures.

	Baseline	During intervention	Post intervention	3-month follow-up
Demographics	Yes			
GAD-7^a^	Yes	At the completion of each module	Yes	Yes
SCAS^b^	Yes	At the completion of each module	Yes	Yes
PedsQL^c^	Yes		Yes	
SUS^d^			Yes	
Online tracking		As used		
Semi-structured questionnaire			Yes	Yes

^a^GAD-7: Generalized Anxiety Disorder, 7-item.

^b^SCAS: Spence Children’s Anxiety Scale.

^c^PedsQL: Pediatric Quality of Life Inventory.

^d^SUS: System Utility Scale.

**Table 2:** Timelines of the study.

Phase	Tasks	Timeframe
Phase 1	Ethics approval, securing of funding	September 2016 to December 2016
	Prototype development via co-design with young people and in conjunction with software company and engineers	January 2017 to December 2017
Phase 2	Recruitment of participants for open trial	September 2017 to December 2017
	Open trial	January 2018 to June 2018
	Analysis of results, write-up, and dissemination of study results	July 2018 to December 2018

### Timeline

The expected timelines of the study are shown in Table 2.

### Equipment Required

An initial working CBT-based prototype intervention will be created in conjunction with a software developer and computer engineer. Biofeedback will be applied via a crude and cost-effective hybrid (most likely an existing HRV-based device that is not fully integrated into the new CBT-based intervention, but used with it). Study participants will be loaned an iPad or similar device on which the necessary technology and analysis software has been pre-loaded.

### Ethics and Consent

This study received ethics approval from the New Zealand Health and Disability Ethics Committee (16/CEN/136) on the 30th of September 2016. Invitations to the co-design focus groups and pilot study will be forwarded to potential participants through clinicians at Starship Hospital to minimize coercion by direct approach. Consent will be obtained directly for those over 16 years of age and via their parents with participant assent for those under 16 years of age. Participants will be free to discontinue engagement at any stage without consequence and this will be made clear to them. Should any unanticipated distress occur during participation, immediate referral will be undertaken to the hospital-based pediatric consult liaison (mental health) team. Data will be presented in a de-identified manner and will be securely stored for 10 years as per University of Auckland regulations.

## Results

Participant recruitment for the first phase of this study commenced in January 2017 and recruitment of participants for the second phase of the study will commence in September 2017. Completion of recruitment is anticipated to occur in June 2018 and analysis of results will be undertaken by December 2018.

## Discussion

### Principal Findings

Anxiety disorders are among the top causes of disability adjusted life years (DALYs) in New Zealand [34] and are the most common type of mental disorder of childhood with a prevalence of 11% in international cohorts [35]. Based on 2013 New Zealand census data [36], we estimate there are over 400,000 young people aged 12 to 18 years in New Zealand. If 10% of them have a long-term physical condition, and up to 40% of this cohort is at risk of an anxiety disorder, that means that over 16,000 children in New Zealand could directly benefit from an intervention that is specifically designed for their needs.

Following the completion of this study, the prototype new game-based eHealth intervention will be refined into a final version for testing in a randomized controlled trial (RCT). If shown in this subsequent study to be clinically effective and acceptable in its final form, it is hoped that the intervention may be hosted on a national eHealth platform and will be made available free of charge to young people in New Zealand. This is currently the case with SPARX, a computerized intervention for depression, but this is contingent on funding from the Ministry of Health.

### Conclusion

The potential health impact of this intervention includes improved access to an acceptable and evidence-based treatment for anxiety in young people with long-term physical conditions and an improvement in the management of both psychological and physical conditions. The potential social impact of such an intervention includes improved functioning for young people with long-term physical conditions by means of reduced school absence, improved social integration, and better relationships with family and clinical teams.

Short-term, the potential economic impact of such an intervention includes reduced cost of intervention (compared to face to face treatment) and reduced parental time off work. Long-term, direct improvements in psychological welfare and indirect improvements in physical welfare are likely to lead to improved chances of completed education and employment. From a service delivery point of view, this intervention could address current resource limitations of mental health services to address the needs of young people with long-term physical conditions.

## Abbreviations

CBT: cognitive behavior therapy
eHealth: electronic health
GAD-7: Generalized Anxiety Disorder, 7-item
HRV: heart rate variability
PedsQL: Pediatric Quality of Life Scale
SCAS: Spence Children’s Anxiety Scale
SPARX: Smart, Positive, Active, Realistic, X-Factor Thoughts
SUS: System Usability Scale

## References

[1] Eiser C (1997). Effects of chronic illness on children and their families. Adv Psychiatr Treat.

[2] Burkhart PV, Dunbar-Jacob JM, Fireman P, Rohay J (2002). Children's adherence to recommended asthma self-management. Pediatr Nurs.

[3] Perrin JM, Bloom SR, Gortmaker SL (2007). The increase of childhood chronic conditions in the United States. JAMA.

[4] van der Lee JH, Mokkink L, Grootenhuis M, Heymans HS, Offringa M (2007). Definitions and measurement of chronic health conditions in childhood: a systematic review. JAMA.

[5] Halfon N, Newacheck PW (2010). Evolving notions of childhood chronic illness. JAMA.

[6] Pless IB, Roghmann KJ (1971). Chronic illness and its consequences: observations based on three epidemiologic surveys. J Pediatr.

[7] Cadman D, Boyle M, Szatmari P, Offord DR (1987). Chronic illness, disability, and mental and social well-being: findings of the Ontario Child Health Study. Pediatrics.

[8] Newacheck PW, McManus MA, Fox HB (1991). Prevalence and impact of chronic illness among adolescents. Am J Dis Child.

[9] Rosenbaum P (1988). Prevention of psychosocial problems in children with chronic illness. CMAJ.

[10] Pao M, Bosk A (2011). Anxiety in medically ill children/adolescents. Depress Anxiety.

[11] Marin T, Chen E, Munch T, Miller GE (2009). Double-exposure to acute stress and chronic family stress is associated with immune changes in children with asthma. Psychosom Med.

[12] Denny S, de SM, Fleming T, Clark T, Merry S, Ameratunga S, Milfont T, Farrant B, Fortune SA (2014). The prevalence of chronic health conditions impacting on daily functioning and the association with emotional well-being among a national sample of high school students. J Adolesc Health.

[13] Juster R, McEwen B, Lupien S (2010). Allostatic load biomarkers of chronic stress and impact on health and cognition. Neurosci Biobehav Rev.

[14] Lewis M, Vitulano LA (2003). Biopsychosocial issues and risk factors in the family when the child has a chronic illness. Child Adolesc Psychiatr Clin N Am.

[15] Sawyer M, Antoniou G, Toogood I, Rice M (1997). Childhood cancer: a two-year prospective study of the psychological adjustment of children and parents. J Am Acad Child Adolesc Psychiatry.

[16] Greenberg PE, Sisitsky T, Kessler RC, Finkelstein SN, Berndt ER, Davidson JR, Ballenger JC, Fyer AJ (1999). The economic burden of anxiety disorders in the 1990s. J Clin Psychiatry.

[17] Opolski M, Wilson I (2005). Asthma and depression: a pragmatic review of the literature and recommendations for future research. Clin Pract Epidemiol Ment Health.

[18] Ramsawh H, Raffa S, Edelen M, Rende R, Keller M (2009). Anxiety in middle adulthood: effects of age and time on the 14-year course of panic disorder, social phobia and generalized anxiety disorder. Psychol Med.

[19] Pine D, Cohen P, Gurley D, Brook J, Ma Y (1998). The risk for early-adulthood anxiety and depressive disorders in adolescents with anxiety and depressive disorders. Arch Gen Psychiatry.

[20] Bennett S, Shafran R, Coughtrey A, Walker S, Heyman I (2015). Psychological interventions for mental health disorders in children with chronic physical illness: a systematic review. Arch Dis Child.

[21] Compton SN, Peris TS, Almirall D, Birmaher B, Sherrill J, Kendall PC, March JS, Gosch EA, Ginsburg GS, Rynn MA, Piacentini JC, McCracken JT, Keeton CP, Suveg CM, Aschenbrand SG, Sakolsky D, Iyengar S, Walkup JT, Albano AM (2014). Predictors and moderators of treatment response in childhood anxiety disorders: results from the CAMS trial. J Consult Clin Psychol.

[22] Chisholm D, Flisher AJ, Lund C, Patel V, Saxena S, Thornicroft G, Tomlinson M, Lancet Global Mental Health Group (2007). Scale up services for mental disorders: a call for action. Lancet.

[23] Merry SN, Stasiak K, Shepherd M, Frampton C, Fleming T, Lucassen MFG (2012). The effectiveness of SPARX, a computerised self help intervention for adolescents seeking help for depression: randomised controlled non-inferiority trial. BMJ.

[24] Holmes J, March S, Spence S (2009). ?Use of the internet in the treatment of anxiety disorders with children and adolescents. ? Counselling, Psychotherapy, and Health 5.1 (2009).

[25] Thabrew H, Stasiak K, Garcia-Hoyos V, Merry SN (2016). Game for health: how eHealth approaches might address the psychological needs of children and young people with long-term physical conditions. J Paediatr Child Health.

[26] Schoenberg P, David A (2014). Biofeedback for psychiatric disorders: a systematic review. Appl Psychophysiol Biofeedback.

[27] Knox M, Lentini J, Cummings TS, McGrady A, Whearty K, Sancrant L (2011). Game-based biofeedback for paediatric anxiety and depression. Ment Health Fam Med.

[28] Culbert TP, Kajander RL, Reaney JB (1996). Biofeedback with children and adolescents: clinical observations and patient perspectives. J Dev Behav Pediatr.

[29] Schwartz MS, Andrasik F (2003). Biofeedback, Third Edition: A Practitioner's Guide.

[30] Brooke J, Jprdam PW, Thomas B, McClelland IL, Weerdmeester B (1996). SUS: a quick and dirty usability scale. Usability Evaluation in Industry.

[31] Spitzer RL, Kroenke K, Williams JB, Löwe B (2006). A brief measure for assessing generalized anxiety disorder: the GAD-7. Arch Intern Med.

[32] Spence SH, Barrett PM, Turner CM (2003). Psychometric properties of the Spence Children's Anxiety Scale with young adolescents. J Anxiety Disord.

[33] Varni JW, Seid M, Kurtin PS (2001). PedsQL 4.0: reliability and validity of the Pediatric Quality of Life Inventory version 4.0 generic core scales in healthy and patient populations. Med Care.

[34] Ministry of Health (2001). The Burden of Disease and Injury in New Zealand.

[35] Kazak AE, Wiener LS, Pao M, Kupst MJ, Farkas Patenaude A (2009). Pediatric Psycho-Oncology: A Quick Reference on the Psychosocial Dimensions of Cancer Symptom Management.

[36] (2001). New Zealand Census Data.

[37] Wiener L, Pao M, Kazak AE, Kupst MJ, Farkas Patenaude A, Holland JC (2009). Quick Reference for Pediatric Oncology Clinicians: The Psychiatric and Psychological Dimensions of Pediatric Cancer Symptom Management.

[38] New Zealand Government (2012). New Zealand Census Data.

